# Reliability of Low-Cost Near-Infrared Spectroscopy in the Determination of Muscular Oxygen Saturation and Hemoglobin Concentration during Rest, Isometric and Dynamic Strength Activity

**DOI:** 10.3390/ijerph17238824

**Published:** 2020-11-27

**Authors:** Claudia Miranda-Fuentes, Isabel María Guisado-Requena, Pedro Delgado-Floody, Leonidas Arias-Poblete, Alejandro Pérez-Castilla, Daniel Jerez-Mayorga, Luis Javier Chirosa-Rios

**Affiliations:** 1 Department Physical Education and Sports, Faculty of Sport Sciences, University of Granada, 18011 Granada, Spain; cmiranda@unab.cl (C.M.-F.); alexperez@ugr.es (A.P.-C.); lchirosa@ugr.es (L.J.C.-R.); 2 Department of Nursing, Physiotherapy and Occupational Therapy, Faculty of Nursing, Group of Preventive Activities in the University Health Sciences Setting, University of Castilla-La Mancha (Universidad de Castilla-La Mancha/UCLM), 02071 Albacete, Spain; IsabelM.Guisado@uclm.es; 3 Department of Physical Education, Sports and Recreation, Universidad de La Frontera, 4811230 Temuco, Chile; pedro.delgado@ufrontera.cl; 4 Faculty of Rehabilitation Sciences, Universidad Andres Bello, 7591538 Santiago, Chile; leonidas.arias@unab.cl

**Keywords:** strength training, dynamometer, tissue saturation, hemoglobin

## Abstract

Background: The objective of this study was to establish the reliability of the Humon Hex near-infrared reflectance spectroscopy (NIRS) in determining muscle oxygen saturation (SmO_2_) and hemoglobin concentration (Hgb) at rest and during isometric and dynamic strength exercises using a functional electromechanical dynamometer (FEMD). Methods: The SmO_2_ and Hgb values of sixteen healthy adults (mean ± standard deviation (SD): Age = 36.1 ± 6.4 years) were recorded at rest and during isometry (8 s), dynamic strength I (initial load of 40% of the average isometric load, with 2 kg increments until muscle failure) and dynamic strength II (same as I, but with an initial load of 40% of the maximum isometric load) activity. To evaluate the reliability in the determination of SmO_2_ and Hgb of this device, intraclass correlation coefficient (ICC), standard error of measurement (SEM) and coefficient of variation (CV) were obtained. Results: The main results obtained are SmO_2_ at rest (CV = 5.76%, SEM = 3.81, ICC = 0.90), isometric strength (CV = 3.03%, SEM = 2.08, ICC = 0.92), dynamic strength I (CV = 10.6, SEM = 7.17, ICC = 0.22) and dynamic strength II (CV = 9.69, SEM = 6.75, ICC = 0.32); Hgb at rest (CV = 1.97%, SEM = 0.24, ICC = 0.65), isometric strength (CV = 0.98%, SEM = 0.12, ICC = 0.96), dynamic strength I (CV = 3.25, SEM = 0.40, ICC = 0.54) and dynamic strength II (CV = 2.74, SEM = 0.34, ICC = 0.65). Conclusions: The study shows that Humon Hex is a reliable device to obtain SmO_2_ and Hgb data in healthy adult subjects at rest and during isometric strength, providing precision for measurements made with this device.

## 1. Introduction

Currently, in the world of sports, knowing the response of skeletal muscle tissue is essential to monitor the behavior of the tissue to physical exercise. [1]. The most used variables to measure sports performance in our days are heart rate, blood lactate concentration and maximal oxygen uptake [2], all of which are mainly systemic parameters. There is little information related to the specific muscle response during physical exercise [2,3]. Increased intramuscular mechanical pressure has been reported to occur during dynamic strength training, leading to a reduction in the blood flow resulting in transient muscle hypoxia that may affect the response of this tissue during physical exercise [4], causing early recruitment of fast twitch fibers due to early fatigue [5].

In order to attain a functional understanding of the muscle response, muscle oxygen saturation (SmO_2_) and hemoglobin concentration (Hgb) have been suggested as values to study [6]. These variables require a localized measurement of muscle response, which can be obtained in a continuous, non-invasive manner using near-infrared spectroscopy (NIRS) methods [6,7,8]. NIRS-based SmO_2_ provides, in a non-invasive way, information on changes in oxygenation and hemodynamics in muscle tissue as a function of the characteristics dependent on the oxygen reflected by the light provided by the NIRS. The result achieved will be the weighted average of hemoglobin oxygen saturations in the vascular bed (small arteries, arterioles, capillaries, venules and small veins) and the myoglobin heme group in muscle fibers [6]. Despite the above, the muscle volume measured by the different NIRS [9], the diversity of devices, the inherent limitations reviewed in the literature regarding these technologies [10], the general lack of standardization of protocols and the determination of validity and reliability of some NIRS remains controversial in the literature.

Of all NIRSs, the most traditional ones involve the use of expensive, cumbersome equipment with limited use in sports practice studies [11,12], and they provide information on the balance between oxygen supply and demand through the concentration of oxygenated (HbO_2_) and deoxygenated hemoglobin (Hb) in the muscle [13]. Technically, these instruments illuminate the muscle with infrared light and detect the light reflected through it, measuring the amount of light that is absorbed, thus providing the values for oxyhemoglobin (HbO_2_), deoxyhemoglobin (HHb), total hemoglobin (tHb) (tHb = HbO_2_ + HHb) and muscle oxygen saturation (SmO_2_ = HbO_2_/tHb), which is expressed as a percentage [14,15,16,17].

Regarding NIRS and physical exercise, Perrey et al. [6] have published a systematic review of the SmO_2_ response in different sport training modalities. In their work, only two articles include this variable with muscle strength training but using an ischemic occlusion technique. Additionally, other researchers have recently published works related to these two variables [18,19,20], and all of them agree that, when the muscles work with greater intensity, more oxygen is consumed and the SmO_2_ decreases [6,21].

In light of the importance of NIRS devices in the determination of SmO_2_, smaller, safe and non-invasive NIRS have been developed to evaluate SmO_2_ in real time, at a relatively low cost. Those devices analyze data using wireless technology, and their validity has been reported both at rest and during exercise [11,22]. Recently, a low-cost NIRS called Humon Hex has been marketed in the field of sports and exercise science. The validity and precision of this NIRS was published by Parisa Farzam et al. [14]; however, although the literature has identified it as a functional and valid instrument, its reliability in the evaluation and training of dynamic strength has not yet been reported [23]. That information is relevant in the field of physical activity and rehabilitation to effectively and reliably monitor one aspect of the muscle response (SmO_2_) to strength training, especially its related to the onset of muscle fatigue due to the association of this variable with the decrease of SmO_2_ during exercise [24], which has shown a significant correlation with the maximum stable state of lactate and the critical power [25] optimizing the physical performance of the individual and their exact needs. [26,27].

The aim of the present study is to determine the reliability of Humon Hex to calculate SmO_2_ and Hgb values at rest and during isometric and dynamic strength activities using a functional electromechanical dynamometer (FEMD).

## 2. Materials and Methods

### 2.1. Subjects

Initially, twenty-three subjects were recruited for the study (five women and eighteen men) of which seven subjects (five women and two men) were excluded because it was not possible to read the SmO_2_ and Hgb record on the device; coincidentally the subjects had the highest body fat percentage, which has been declared as a limitation for reading these devices [9]. Finally, sixteen healthy men volunteered to participate in this study (mean ± standard deviation (SD): Age = 36.12 ± 6.39 years, body mass = 80.37 ± 10.03 kg, body height = 1.73 ± 0.06 m, body mass index = 26.65 ± 2.73 kg/m^2^, body fat percentage = 23.45 ± 4.85%, skeletal muscle mass = 34.97 ± 3.69 kg and skeletal muscle mass of the lower limb = 18.64 ± 2.17 kg). Selective inclusion criteria included (I) having at least one year of experience in strength training and (II) being free of medical comorbidities or recent musculoskeletal injuries that could compromise physical performance.

### 2.2. Ethics

All subjects were informed of the procedures to be used and signed a written informed consent form before initiating their participation in the study. The study protocol adhered to the tenets of the Declaration of Helsinki and was approved by the University of Granada Institutional Review Board (IRB approval: 997/CEIH/2019).

### 2.3. Procedures

**Figure 1:**
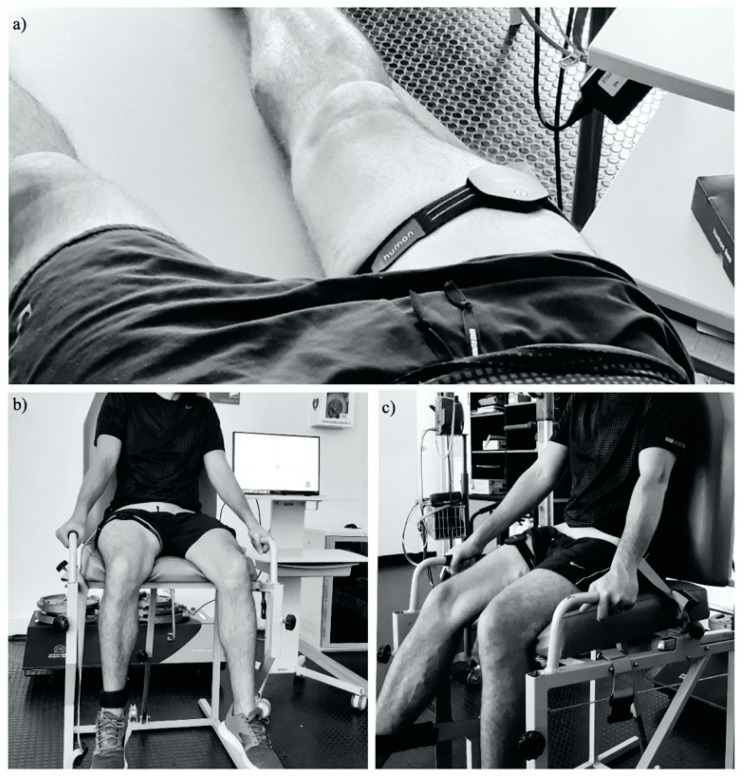
Graphic representation of near-infrared spectroscopy (NIRS) positioning and assessment of dynamic strength with muscle oxygen saturation (SmO_2_) and hemoglobin concentration (Hgb) registration. (**a**) rest; (**b**) seated position with knee in 90° flexion for isometric strength; and (**c**) seated position for knee extension, executing dynamic strength I and II.

Subjects attended two familiarization sessions, 48–72 h apart, one week before the first experimental session. For each experimental session, the participants were invited to the laboratory after a four-hour fast, rested and without having consumed caffeine 24 h before the experimental session. All evaluations were carried out at the university research laboratory at the same time of the day for each subject (±1 h) and under similar environmental conditions (≈22 °C and ≈60% of humidity). The first experimental session (test) began with the collection of body composition data. Subsequently, Humon Hex was placed at the midpoint of the thigh of the dominant leg, between the anterior-superior iliac spine and the upper edge of the patella, and was securely positioned with a velcro fastener [15] (Figure 1). Once the device was affixed, each subject had a 15-min rest period lying on their backs on a stretcher (Figure 1a). After the rest period, all participants performed a standard warm-up on a low-load ergometer (modified Borg scale <2–3) at a speed of 60 rpm for 10 min and, subsequently, proceeded to the dynamic strength protocols.

The second experimental session (retest) was carried out 48 h after the test moment, all the protocols that registered SmO_2_ and Hgb in the first session were repeated, such as resting time, warm-up and all the dynamic strength protocols.

#### 2.3.1. Dynamic Strength Protocols

**Figure 2:**
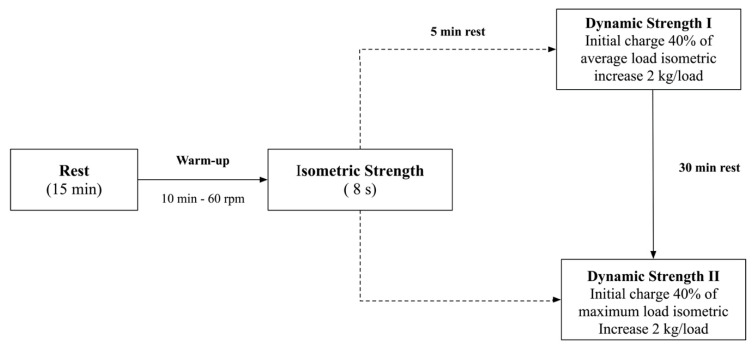
Scheme of protocols to assess isometric and dynamic strength.

For all dynamic strength protocols, participants sat in a specially designated chair and fixed a pulley in the distal area of their dominant leg to provide the FEMD values (Figure 1b,c). Three protocols were carried out for strength evaluations: Isometric strength, dynamic strength I and dynamic strength II. The first measurement was isometric strength. Each subject was asked to perform three 8-s sets of maximum isometric strength, with a 60-s rest between each series; the instruction to each subject was to perform knee extension at the feasible maximum intensity. In this protocol, the average load and the maximum load of each repetition were recorded. After a five-minute rest, the subjects performed the dynamic strength I protocol, which required the application of maximum strength with an initial load of 40% of the average load obtained in the isometric strength. The dynamic strength II measurement required the application of maximum strength with an initial load of 40% of the maximum load obtained in the isometric strength measurement. The instructions to perform each of the dynamic force protocols were to perform knee extensions up to 180°, which would have load increases of two kilograms (kg) from the first repetition until attaining muscle failure. Between each dynamic strength protocol, the participants had a 30-min rest (Figure 2).

#### 2.3.2. Equipment

A stadiometer was used to measure body height (Seca 202; Seca Ltd., Hamburg, Germany) and a bioimpedaniometer was used to measure body composition (InBody 770, Cerritos, CA, USA). SmO_2_ and Hgb were recorded using a NIRS (Humon Hex, Dynometrics Inc., Boston, MA, USA). This is a 60.5 by 57 by 13.8 mm device with a weight of 32 g. Its plastic case is slightly curved to allow easy contact with the quadricep surface. Finally, dynamic strength was assessed using a FEMD (Dynasystem, Model Research, Granada, Spain), which was reported to be a valid and reliable instrument to assess this ability in the upper [23,28,29] and lower limbs [30].

#### 2.3.3. Data Extraction

Humon Hex links, via Bluetooth, to a smartphone in which a personalized application (Dynamometrics Inc, Boston, MA, USA, retrieved from http://humon.io) shows the progress of the training in real time. The Humon only measures light intensity at three separations and two wavelengths, therefore, to estimate its results, it is necessary to assume a fixed dispersion coefficient. The algorithms used to determine the hemodynamic parameters have not been published and are the property of Dynometrics Inc. [14]. The total data recorded for each experimental session were extracted to a spreadsheet from the Humon Hex website. In the registration form of each participant, the start and finish seconds for each protocol obtained from the mobile application were written down. For the analysis of the SmO_2_ and Hgb variables, the average value obtained from each isometric and dynamic strength protocol was used. In the case of the data extracted from the FEMD, they were exported from the device to a customized spreadsheet using the average and maximum values for their subsequent analysis.

### 2.4. Statistical Analyses

Descriptive data are presented as mean ± SD. The normal distribution of the data was confirmed using the Shapiro–Wilk test (*p* > 0.05). Paired sample t-test and standardized mean differences (Cohen’s effect size (ES)) were used to compare the magnitude of the load, number of repetitions, SmO_2_, and Hgb between both testing sessions. The criteria to interpret the magnitude of the ES were as follows: Null (<0.20), small (0.2–0.59), moderate (0.60–1.19), large (1.20–2.00) and very large (>2.00) [31]. Absolute reliability was assessed using the standard error of measurement (SEM) and coefficient of variation (CV), while relative reliability was assessed using the ICC, model 3.1. The following criteria were used to determine acceptable (CV ≤ 10%, ICC ≥ 0.80) and high (CV ≤ 5%, ICC ≥ 0.90) reliability [32]. The ratio between two CVs was used to compare the reliability of the SmO_2_ and Hgb between the different experimental conditions. The smallest important ratio of CVs was considered to be higher than 1.15 [33]. Systematic bias were examined through Bland–Altman plots. Finally, the Pearson’s product-moment correlation coefficient (Pearson’s r) was used to quantify the correlation of SmO_2_ and Hgb between both testing sessions. The criteria to interpret the magnitude of the r were null (0.00–0.09), small (0.10–0.29), moderate (0.30–0.49), large (0.50–0.69), very large (0.70–0.89), nearly perfect (0.90–0.99) and perfect (1.00) [31].

For all statistical calculations, a 95% confidence interval was used in the analysis. Statistical significance was accepted at *p* < 0.05. All reliability assessments were performed by means of a customized spreadsheet [34], while other statistical analyses were performed using the JASP software (version 0.9.1.0, http://www.jasp-stats.org).

## 3. Results

No significant differences were observed for the average load during the isometric and dynamic strength conditions (*p* = 0.462; ES = 0.18) as well as for the maximum load and number of repetitions during the dynamic strength condition following protocol I (*p* = 0.249; ES = 0.29) between both testing sessions. No significant or small to moderate differences were observed for the remaining comparisons between both testing sessions (*p* = 0.074; ES = 0.47) (Table 1).

**Figure 3:**
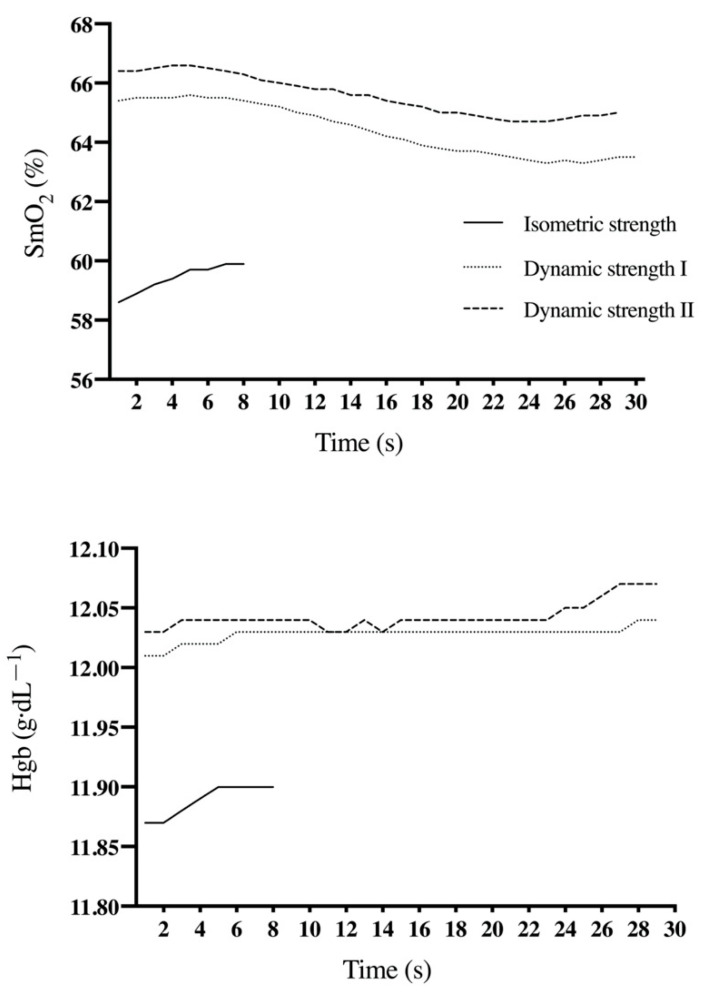
Behavior of SmO_2_ and Hgb in a representative individual at rest and during isometric strength and dynamic strength conditions.

**Figure 4:**
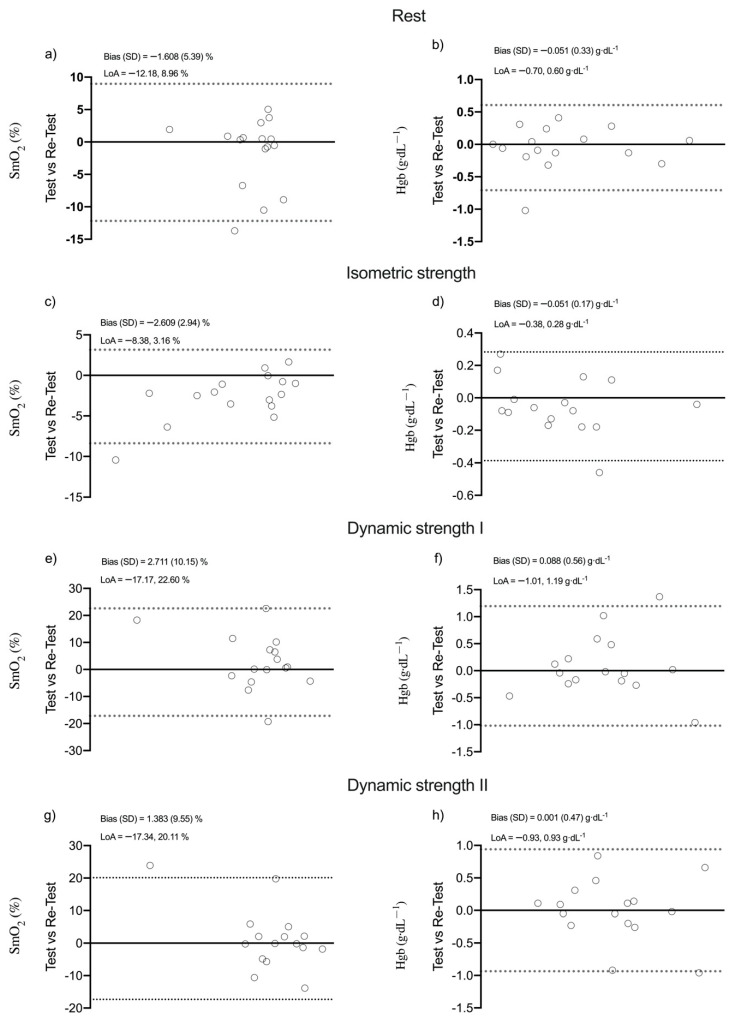
Bland-Altman plots of test-retest for SmO_2_ (%) and Hgb (g·dL^−1^). (**a**) SmO_2_ (%) at rest, (**b**) HgB (g·dL^−1^) at rest, (**c**) SmO_2_ (%) at isometric strength (**d**) HgB (g·dL^−1^) at isometric strength, (**e**) SmO_2_ (%) at dynamic strength I, (**f**) HgB (g·dL^−1^) at dynamic strength I, (**g**) SmO_2_ (%) at dynamic strength II, (**h**) HgB (g·dL^−1^) at dynamic strength II.

Figure 3 shows the behavior of SmO_2_ and Hgb in a representative individual under the different experimental conditions. No significant differences were found for both SmO_2_ and Hgb during the different experimental conditions between both testing sessions (*p* = 0.249; ES = 0.34), except for SmO_2_ under isometric strength condition (*p* = 0.002; ES = 0.38). Absolute reliability was from acceptable to high for SmO_2_ (SEM = 6.75%, CV = 9.69%) and high for Hgb (SEM = 0.40 g·dL^−1^, CV range = 3.25%) in the different experimental conditions, except for SmO_2_ under dynamic strength conditions following protocol I (SEM = 7.17%, CV = 10.6). Relative reliability was high for SmO_2_ under rest and isometric strength conditions (ICC range = 0.90–96) as well as for Hgb under isometric strength conditions (ICC = 0.96), while unacceptable relative reliability was observed in the remaining conditions (ICC = 0.65) (Table 2). Regarding reliability comparisons, (I) the isometric strength condition provided the SmO_2_ and Hgb both higher reliability than the rest and dynamic strength conditions (CVratio = 1.90), (II) the rest condition provided the SmO_2_ and Hgb with higher reliability than the dynamic strength conditions (CVratio = 1.39) and, finally, (III) the dynamic strength condition following protocol II provided the Hgb with higher reliability than the dynamic strength condition following protocol I (CVratio = 1.19). Bland–Altman plots reveals a higher systematic bias for SmO_2_ during the dynamic strength II protocol (Figure 4).

**Figure 5:**
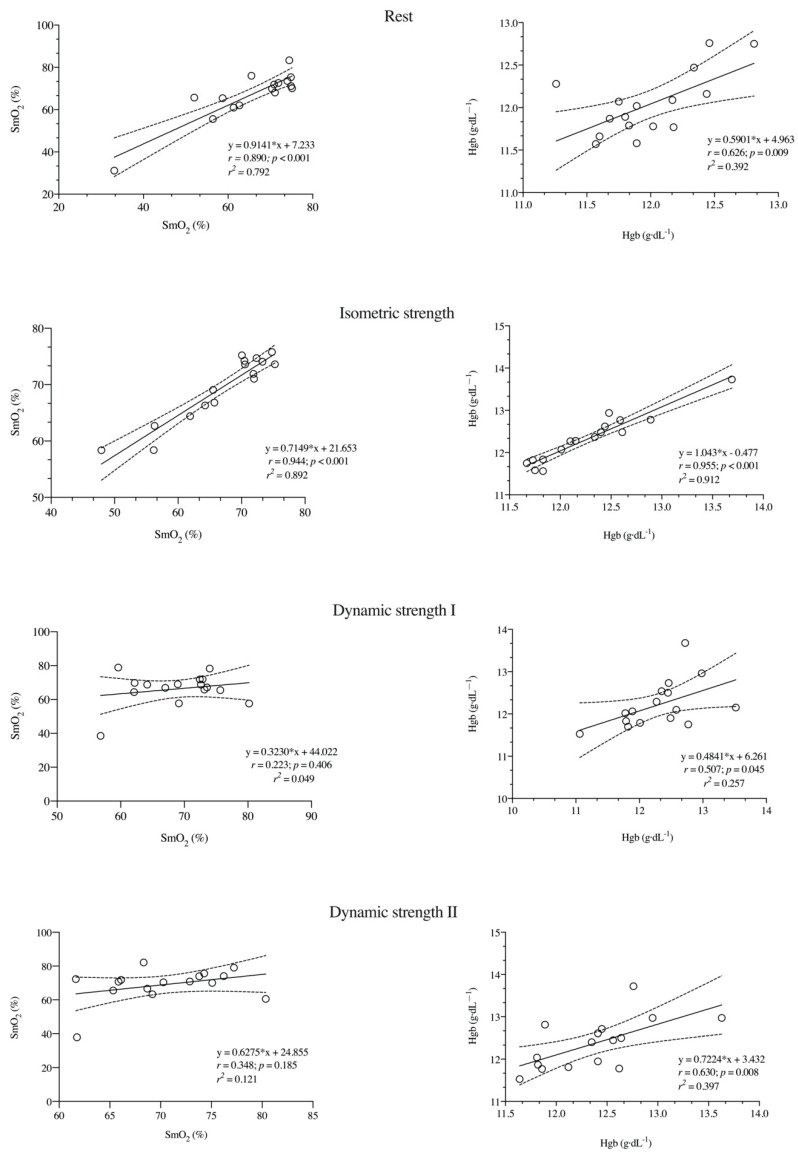
Relationship between SmO_2_ and Hgb at rest and during isometric strength and dynamic strength (protocols I and II) conditions between both testing sessions.

Finally, the r magnitude for SmO_2_ and Hgb was from large to nearly perfect during the different experimental conditions (*r* = 0.507; *p* = 0.045), except for SmO_2_ under dynamic strength conditions (*r* range = 0.348; *p* = 0.185) (Figure 5).

## 4. Discussion

The objective of this study was to establish the reliability of the Humon Hex NIRS in determining muscle oxygen saturation and hemoglobin concentration at rest and during isometric and dynamic strength exercises using a functional electromechanical dynamometer. The main findings of this study suggest that this NIRS is reliable in rest and isometry conditions as it displays stable repeatability (CV < 5.76%), which becomes less accurate under increasing intensity and muscular strength (dynamic) exercise. 

Previous studies that approached SmO_2_ using other NIRS positioned in the vastus lateralis during a dynamic maximum knee extension exercise obtained results that were very similar to those obtained in this study, revealing that the knee extension exercise caused a slight increase in this variable and then a decreasing trend according to all the publications consulted [19,35,36,37]. On the other hand, Gómez–Carmona et al. [20], McManus et al. [38] and Davis et al. [18] evaluated the SmO_2_ and Hgb responses in other dynamic strength protocols using other NIRS and explained that, as effort intensity increases, SmO_2_ tends to decrease. Regarding SmO_2_ and Hgb’s behavioral tendency published in the literature on Humon Hex, we have only found one publication about an incremental resistance exercise using a stationary ergometer with behavioral results equivalent to those obtained in this study [14].

Furthermore, regarding the response of SmO_2_ to the isometry protocol, our results are different from those obtained in other studies that measured SmO_2_ with NIRS placed on the vastus lateralis during the exercise of maximum isometric extension of the knee, in which an immediate decrease in SmO2 at the start of the isometric contraction was observed versus an initial reaction towards the increase in the variable of this study [38,39,40]. It must be noted that the isometry protocols reviewed in the literature consisted of a total of 20 to 30 s until exhaustion, differing from the 8 s used in the present study, which could be insufficient for the subject’s effort. Despite this, our results are consistent with those of one individual in the study by Pereira et al. [40], where SmO_2_ increased during the first seconds of the exercise.

As far as our review goes, this is the first study that sought to assess the Humon Hex accuracy during dynamic and isometric muscular strength exercises. The literature has stated the reliability of other NIRS during muscular strength exercises [41] and under other experimental circumstances of physical exercise. The reliability results have been similar to those obtained in this study, such as incremental cycling (ICC = 0.77–0.99) [11], high-intensity cycling exercise (ICC = 0.67) [42], orthostatic challenge (ICC = 0.75) [43] and arterial occlusion method, passive tests and active test [36].

From a physiological point of view, the response of skeletal muscle to the effort achieved in this study can be justified because the consumption of oxygen at the muscular level exceeded its supply, which theoretically increases blood lactate levels and is the response to an acceleration of glycolysis on the supply of oxidative energy [14]. This phenomenon has been explained like this: When the muscle oxygen uptake suddenly increases in proportion to the increases in motor unit recruitment and muscle fiber activation, a quick way to increase the oxygen supply is to improve the removal of hemoglobin from the blood flowing through the tissue. In general, in a muscle at rest, the oxygen extraction is between 20%–40% [44], which increases to 70%–80% in rising levels of exercise [45], which could explain the decrease in SmO_2_ during physical effort.

### 4.1. Strengths and Limitations

This study was not without limitations; the SmO_2_ and Hgb responses were only assessed in the dominant lower limb. It would be interesting to assess both segments to contrast and describe metabolic behaviors. Furthermore, a decision was made to monitor only the rectus femoris instead of another muscle as other publications deemed it to be the most common area to examine SmO_2_ with NIRS [6,14]; however, it would be interesting to extend the description to other muscle segments. Furthermore, an evaluation of the venous hemoglobin concentration would have helped to confirm consistent conditions and will be considered in future investigations. It must be noted that this study is the first to use Humon Hex in dynamic strength exercises, so it is not possible to contrast our results with other publications about the assessment of this device. Future research could increase the sample size and include women and other age ranges to establish normative values for SmO_2_ and Hgb. In addition, perform the validity of this device in the same conditions of the strength protocols proposed for this study showing the comparison with the standard gold device.

### 4.2. Clinical Application

The proposed results will make it possible to apply this NIRS device and learn with more reliability about essential aspects of the metabolic behavior of muscle tissue related to SmO_2_ in conditions of rest and isometric strength, enhancing the functional and non-invasive utilities of Humon Hex. Consequently, the trainer could have an additional tool to know the metabolic response of muscle tissue in protocols similar to those proposed at rest and isometry, monitoring aspects related to the onset of muscle fatigue due to the association of this variable with the decrease in SmO_2_ during physical exercise [24], which could be helpful for the individual’s physical performance and exact needs.

## 5. Conclusions

The findings of this study demonstrate that Humon Hex is a reliable instrument to measure changes in the local characteristics of oxygen in muscle hemoglobin, which makes it a useful device to monitor at rest and during isometric exercise activities, proving unreliable for more dynamic exercises.

## Figures and Tables

**Table 1:** Comparison of the average load, maximum load and number of repetitions during the isometric and dynamic (protocol I and II) strength conditions between both testing sessions.

Condition	Variable	Session 1 (Mean ± SD)	Session 2 (Mean ± SD)	*p*-Value	ES (95% CI)
Isometric strength	Average load (kg)	50.7 ± 9.6	49.7 ± 9.1	0.545	0.15 (−0.34, 0.64)
	Maximum load (kg)	58.7 ± 11.8	56.3 ± 9.9	0.074	0.47 (−0.04, 0.99)
Dynamic strength I	Average load (kg)	26.4 ± 7.4	27.0 ± 6.8	0.462	0.18 (−0.68, 0.30)
	Maximum load (kg)	46.6 ± 7.1	47.4 ± 7.4	0.402	0.21 (−0.70, 0.28)
	Number of repetitions	10.2 ± 2.3	10.6 ± 2.3	0.249	0.29 (−0.79, 0.20)
Dynamic strength II	Average load (kg)	29.0 ± 7.6	29.2 ± 7.2	0.792	0.06 (−0.55, 0.42)
	Maximum load (kg)	47.0 ± 9.3	50.0 ± 7.6	0.040	0.56 (−1.08, 0.02)
	Number of repetitions	8.8 ± 2.9	9.8 ± 2.5	0.013	0.69 (−1.23, 0.13)

SD, standard deviation; ES, Cohen’s *d* effect size ([higher mean-lower mean]/SD both); 95% CI, 95% confidence interval.

**Table 2:** Humon Hex NIRS reliability for the determination of SmO_2_ and Hgb at rest, isometric and dynamic strength protocols I and II.

	Condition	Session 1 (Mean ± SD)	Session 2 (Mean ± SD)	*p*-Value	ES (95% CI)	SEM (95% CI)	CV (95% CI)	ICC (95% CI)
SmO_2_ (%)	Rest	65.5 ± 11.3	66.7 ± 11.65	0.251	0.14 (−0.84, 1.12)	3.81 (2.85, 5.90)	5.76 (4.24, 8.91) ^†^	0.90 (0.75, 0.97)
	IS	66.8 ± 7.8	69.4 ± 5.9	0.002	0.38 (−0.61, 1.36)	2.08 (1.54, 3.22)	3.03 (2.24, 4.69) *	0.92 (0.79, 0.97)
	DS I	69.0 ± 6.5	66.3 ± 9.4	0.301	0.34 (−1.32, 0.65)	7.17 (5.30, 11.1)	10.6 (7.83, 16.4)	0.22 (−0.29, 0.64)
	DS II	70.4 ± 5.5	69.1 ± 10.0	0.571	0.17 (−1.14, 0.82)	6.75 (4.99, 10.5)	9.69 (7.15, 15.0)	0.32 (−0.20, 0.69)
Hgb (g·dL^−1^)	Rest	12.0 ± 0.4	12.0 ± 0.4	0.549	0.13 (−0.85, 1.10)	0.24 (0.18, 0.37)	1.97 (1.46, 3.05) ^†^	0.65 (0.25, 0.86)
	IS	12.3 ± 0.5	12.4 ± 0.6	0.249	0.10 (−0.80, 1.16)	0.12 (0.09, 0.19)	0.98 (0.72, 1,51) *	0.96 (0.89, 0.99)
	DS I	12.3 ± 0.6	12.2 ± 0.6	0.541	0.16 (−1.14, 0.81)	0.40 (0.29, 0.62)	3.25 (2.20, 5.04)	0.54 (0.70, 0.81)
	DS II	12.4 ± 0.5	12.4 ± 0.6	0.987	0.00 (−0.98, 0.98)	0.34 (0.25, 0.52)	2.74 (2.02, 4.23) ^#^	0.65 (0.25, 0.86)

SD, standard deviation; ES, Cohen’s d effect size ([higher mean – lower mean]/ SD both); SEM, standard error of measurement; CV, coefficient of variation; ICC, intraclass correlation coefficient; 95% CI, 95% confidence interval. IS, Isometric strength; DS I, Dynamic strength I; DS II, Dynamic strength II; Bold number indicate an inacceptable reliability (CV > 10%, ICC < 0.80). *, significantly more reliable than the rest and dynamic strength conditions; †, significantly more reliable than the dynamic strength conditions; #, significantly more reliable than the dynamic strength condition following protocol I (CVratio [higher CV values/lower CV values] > 1.15).
